# Increased soluble IL-2 receptor levels in serum from a patient with painless thyroiditis

**DOI:** 10.1186/1756-6614-6-12

**Published:** 2013-12-05

**Authors:** Kae Hamamoto, Masaaki Inaba, Shinsuke Yamada, Maki Yoda, Koichiro Yoda, Hitoshi Goto, Masafumi Kurajoh, Hidenori Koyama

**Affiliations:** 1 Department of Nephrology, Department of Metabolism, Endocrinology and Molecular Medicine, Osaka City University Graduate School of Medicine, 1-4-3, Asahi-machi, Abeno-ku, Osaka, 545-8585, Japan; 2 Department of Internal Medicine, Division of Diabetes, Endocrinology and Metabolism, Hyogo College of Medicine, Mukogawa-cho 1-1, Nishinomiya, Hyogo, 663-8501, Japan

**Keywords:** Thyroiditis, sIL-2R, Autoimmune

## Abstract

**Context:**

Serum concentration of soluble interleukin-2 receptor (sIL-2R) has been established as a reliable marker of T-lymphocyte activation. However, there have been no reports describing the relationship between serum sIL-2R and painless thyroiditis.

**Objective:**

We report a case of a 76-yr-old female with a significant and temporary increase of sIL-2R concomitant with painless thyroiditis.

**Case illustration:**

The patient was diagnosed with malignant lymphoma at the age of 73. After 6 cycles of CHOP-R complete remission was induced and no recurrence was observed up to 3.5 years. At 76 years of age, she exhibited hyperthyroidism and was diagnosed with painless thyroiditis based upon US examination and ^99m^Tc-Thyroid scintigraphy. Her AST and ALT were mildly elevated, and her serum level of sIL-2R increased up to 2230 U/mL from the approximately 540 U/mL, which had been stable for 3 years before.

These abnormal data normalized without requiring any treatment. The time-course of the reduction in sIL-2R did not correlate with FT4 or FT3, but was very similar to that of AST and ALT.

**Conclusion:**

There was no evidence of relapse of the malignant lymphoma. We conclude that the increase of sIL-2R was associated with painless thyroiditis. Considering the similar time-course between the reduction of serum sIL-2R and those of AST and ALT, which are often accompanied by autoimmune processes in painless thyroiditis during the development of hyperthyroidism, it was suggested that the increase of serum sIL-2R in this case resulted from activation of an autoimmune process.

## Background

The serum concentration of soluble interleukin-2 receptor (sIL-2R), which is derived from its specific membrane receptor on activated T lymphocytes [1], has been established as a reliable marker of T-lymphocyte activation [2]. In autoimmune hyperthyroidism, such as Graves’ disease, serum sIL-2R likely increases as a result of T cell activation [3]. This hypothesis is supported by the further increase of sIL-2R in patients with refractory Graves’ disease [4]. However, it remains possible that hyperthyroidism alone also increases serum sIL-2R, even in the absence of autoimmune activation, based on the increase in sIL-2R that occurs with triiodothyronine replacement [5]. However, to date, there has been no report on the relationship between serum sIL-2R and painless thyroiditis, which is known as subacute variant of chronic lymphocytic thyroiditis,, which is a clinical syndrome that manifests as transient thyrotoxicosis followed by transient hypothyroidism without spontaneous pain or tenderness in thyroid gland. On histopathologic analysis, painless thyroiditis manifests as a lymphocytic infiltration of the thyroid follicles, resulting in follicular cell damage [6].

Herein, we report a case of a patient who was in remission from large cell type lymphoma with restoration of sIL-2R levels within normal range, and was being periodically monitored for any potential relapse of large cell type lymphoma. She exhibited a significant and temporary increase of sIL-2R, which occurred with the same time-course as painless thyroiditis.

## Case presentation

In January 2007, a 73-year-old female, developed abdominal pain with pyrexia and was diagnosed with acute appendicitis. Examination by computed tomography to further assess the appendicitis detected disseminated enlargement of the retroperitoneal lymph nodes and atrophy of the right kidney surrounded enlargement of multiple lymph nodes. Since she was suspected to be suffering from malignant lymphoma, she was then referred to the Department of Hematology, Osaka City University Hospital on February 7, 2007 for further examination.

She was diagnosed as having malignant lymphoma (follicular lymphoma Grade 3a, Stage 2), and was treated with 6 cycles of CHOP-R, which resulted in complete remission. There was no recurrence for another 3.5 years, and periodic measurements of serum sIL-2R revealed that her serum sIL-2R remained within the normal range; around 500 U/ml. In September 2010, she felt fatigue and palpitation, and subsequently developed excessive perspiration. She was identified as developing hyperthyroidism, as reflected by an increase of serum free T4 and free T3 above the normal upper limit to 3.2 ng/ml and 15.6 pg/ml respectively, with simultaneous suppression of serum TSH to <0.004 mIU/l. Of great importance was the significant increase of sIL-2R up to 2230 U/ml, which had been stable for 3 years around 540 U/ml. Once the potential relapse of malignant lymphoma had been ruled out and treatment for hyperthyroidism had been initiated, she was admitted to Osaka City University Hospital.

She was 152.0 cm in height and 51.0 kg in weight, which did not change appreciable before admission. She did not lose weight. The pulse rate was 72/min and regular, due probably to administration of atenolol at the daily dose of 50 mg, and the blood pressure was 132/68 mmHg with a body temperature of 36.0°C. She had a diffuse and elastic goiter, but she had neither spontaneous pain nor tenderness in the anterior neck area. There was no sign of a palpable cervical lymphoid node and leg edema was absent. The laboratory findings on admission are presented in Table 1. Laboratory tests confirmed primary hyperthyroidism, as indicated by the increase of serum FT4 and FT3 above the respective normal upper limits, with significant suppression of TSH to an undetectable level. She exhibited a positive test for anti-thyroglobulin antibody, but was negative for anti-TSH receptor antibody or anti-thyroid peroxidase antibody. In addition to hyperthyroidism, the abnormal findings detected on admission included a slight reduction of hemoglobin to 10.8 g/dl, mild liver dysfunction (AST 34 IU/l, ALT 36 IU/l), and slight renal dysfunction, likely due to total loss of right renal function as a result of pararenal lymph node enlargement during the onset of malignant lymphoma.

**Table 1:** Laboratory data on admission


**Hematology tests**			eGFR	(90<)	41 ml/min/1.7 m^2^
WBC	(4300–8000)	4700 /μl	CPK	(30–140)	46 IU/l
Neut	(49.5–71.0)	56.1%	Na	(137–146)	136 mEq/l
Mono	(2.3–7.7)	10.0%	K	(3.8-5.1)	4.1 mEq/l
Eos	(0.2–6.8)	2.8%	Cl	(98–108)	105 mEq/l
Baso	(0.0–1.8)	0.4%	Ca	(7.0-10.0)	8.7 mg/dl
Lymph	(26.6–46.6)	30.7%	P	(2.9-4.3)	3.7 mg/dl
RBC	(395–465)	349 × 10^4^/μl	TC	(130–220)	153 mg/dl
Hb	(11.3–14.9)	10.8 g/dl	TG	(50–150)	60 mg/dl
Hematocrit	(36–47)	32.2%	FPG	(70–109)	86 mg/dl
PLT	(18.0–34.0)	19.2 × 10^4^ /μl	CRP	(0–0.4)	0.01 mg/dl
			ESR 1 h	(2–15)	5 mm
**Biochemistry tests**			sIL-2R	(124–466)	2230 U/ml
Total protein	(6.5–8.5)	5.8 mg/dl			
Albumin	(3.5–5.0)	3.9 g/dl	**Thyroid-related tests**		
AST	(13–33)	34 IU/l	FT4	(0.9-1.7)	3.2 ng/dl
ALT	(6–27)	36 IU/l	FT3	(2.3-4.0)	15.6 pg/ml
ALP	(115–359)	189 IU/l	TSH	(0.5-5.0)	<0.004 mIU/l
LDH	(119–229)	195 IU/l	TRAb	(<1.0)	<1.0 IU/l
BUN	(7–18)	25 mg/dl	TPOAb	(<0.3)	<0.3 IU/ml
Creatinine	(0.4–0.9)	1.0 mg/d	TgAb	(<0.3)	3.9 U/ml

Underlined data is abnormal value.

WBC, white blood cell; RBC, red blood cell; Hb, Hemoglobin; PLT, platelet; AST, asparate transaminase; ALT, alanine transaminase; ALP, alkaline phosphatase; LDH, lactate dehydrogenase; BUN, blood urea nitogen; eGFR, estimated glomerular filtration rate; CPK, creatine phosphokinase; TC, total cholesterol; TG, Triglycerides; FPG, fasting plasma glucose; CRP, C-reactive protein; ESR, erythrocyte sedimentation rate; sIL-2R, soluble interleukin-2 receptor; FT4, free thyroxine; FT3, free triiodothyronine; TSH, thyrotropin; TRAb, TSH receptor antibody; TPOAb, thyroid peroxidase antibody; TgAb, Thyroglobulin antibody.

**Figure 1:**
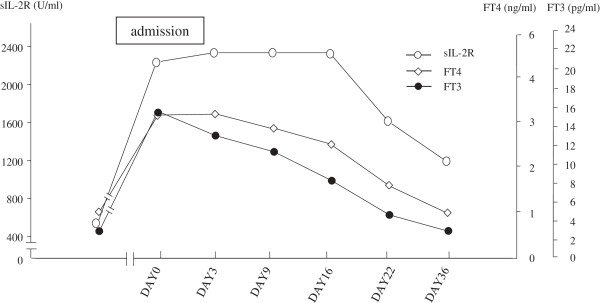
**Time course of the SIL-2R, FT3 and FT4 levels during admission.** FT3 and FT4 were reduced by the 3rd Hospital day Serum sIL-2R persisted at a high level until the 16th Hospital day and began to decline 13 days later; after reduction of serum FT4 and FT3.

**Figure 2:**
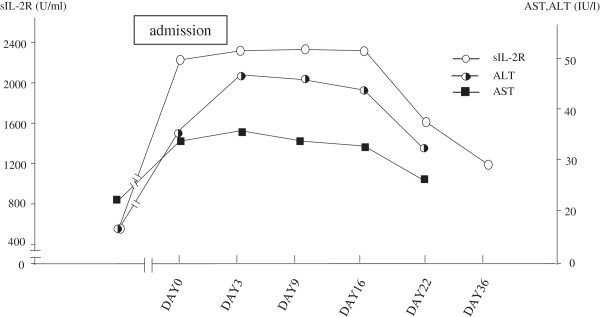
**Time course of SIL-2R, FT3 and FT4 levels during admission.** The reduction of sIL-2R was similar to that of the liver enzymes, AST and ALT.

Ultrasound examination of the thyroid gland showed destructive thyroiditis, as reflected by the apparent reduction and heterogeneity of echogenicity inside the thyroid gland. Intrathyroidal blood flow measured at the inferior thyroid artery was 24.3 cm/s, which was not as high as the mean value of 41.5 ± 26.6 m/sec, observed in untreated Graves’ disease [7]. ^99m^Tc-Thyroid scintigraphy showed essentially no uptake of 0.187% within the thyroid gland. Based on these findings, we diagnosed her etiology of primary hyperthyroidism as painless thyroiditis, and then monitored her thyroid function without any medication. Her serum FT4 and FT3 levels decreased rapidly over time without treatment, whereas her serum sIL-2R exhibited a sustained increase until the 16^th^ Hospital day and started to decrease approximately 7 days later.(Figure 1) Therefore, the reduction of thyroid function was apparently followed by the reduction of serum sIL-2R. Of interest, the time-courses of the reductions in AST and ALT during admission were similar to that of serum sIL-2R (Figure 2). After the reduction of sIL-2R to around 500 U/ml, it was maintained at a stable range. She was discharged from our hospital after 21 Hospital days. When her serum TSH increased above the detectable lower limit to 0.013 mIu/l 35 days after admission, thyroid hormone replacement therapy was initiated at a daily dose of 50 μg L-thyroxine, and this dose was adjusted upward to 125 μg/day.

The hematologist investigated the possible recurrence of large cell type lymphoma, which had been in remission for 3 years before. However, based on her normal findings on chest and abdominal X-ray CT, with no remarkable change of LDH, an indicator of lymphoma activity, the hematologist ruled out the relapse of malignant lymphoma, which was further supported by the lack of new manifestation suggesting its recurrence for another 2 years until now.

## Discussion

Herein, we report a case in which a significant and temporary increase of serum sIL-2R was detected during an episode of painless thyroiditis simultaneously with the temporary increase of serum FT4 and FT3, which was supported by the periodic measurement of serum sIL-2R during ongoing follow-up for the possible relapse of her malignant lymphoma. This case showed apparent reduction of serum FT3 and FT4 by the 3^rd^ Hospital day, although her serum sIL-2R remained at a sustained high level until the 16^th^ Hospital day and started to decline 13 days later, after the reduction of serum FT4 and FT3 (Figure 1).

The reason for the increased sIL-2R in hyperthyroidism is mainly explained by two mechanisms. Hyperthyroidism alone could increase sIL-2R [5,8,9], in addition to the hyper-immune state with T cell auto-activation, which is often associated with hyperthyroidism [5]. Serum sIL-2R has previously been demonstrated to correlate in a positive manner with serum FT3 in non-autoimmune thyroiditis, as well as in autoimmune thyroiditis [5]. Furthermore, serum sIL-2R levels have been shown to be higher in the latter group than in the former; indicating the increase of sIL-2R as a result of increased autoimmunity, in addition to hyperthyroidism [5].

Since the increase of sIL-2R persisted, despite the apparent reduction of serum FT4 and FT3 (Figure 1 above), it was suggested that hyperthyroidism alone could not be responsible for the increased sIL-2R in this patient. Further support of this hypothesis is that serum sIL-2R remained unchanged, around 800 U/ml, even after the reduction of serum FT4 and F3 below the normal lower limit, with a significant increase in serum TSH up to 17 mIu/l, although the hematologist had ruled out the possibility of recurrence of malignant lymphoma.

Another important mechanism that has been proposed to increase serum sIL-2R in hyperthyroidism is activation of the autoimmune process, since serum sIL-2R has been established as a reliable marker of T-lymphocyte activation [1]. In fact, it has previously been reported that hyperthyroid patients with refractory Graves’ disease exhibited higher levels of serum sIL-2R than would be predicted from their hyperthyroid state [10]. Furthermore, it has also been reported that patients with subacute thyroiditis showed increased sIL-2R; although to date, there is no available data regarding the association of painless thyroiditis with increased sIL-2R.

Of great interest, the time course of the reduction in sIL-2R was similar to that of the liver enzymes, AST and ALT, during the episode of painless thyroiditis (Figure 2). It was previously reported that thyroid-liver interaction in autoimmune thyroid disease is partly explained by the association of intrinsic liver disease with intrinsic thyroid disease through autoimmune mechanism(s) [11-13]. Since the reductions of AST and ALT were delayed by 13 days, compared with that of serum FT3 and FT4, the increase of AST and ALT could have occurred by an autoimmune mechanism, in addition to the hepatotoxic effect of excess thyroid hormone. Considering the similar time-course of the reduction of serum sIL-2R with those of AST and ALT during the episode of hyperthyroidism, which is often accompanied by the autoimmune process in painless thyroiditis [12,13], it was suggested that the increase of serum sIL-2R in this case resulted from activation of the autoimmune process during this episode. However, since it is reported that the normalization of hypermetabolic state might be delayed for certain period even after complete normalization of serum FT4 and FT3 within their respective normal range, it could not be completely negated that the increase of sIL-2R might be explained by hyperthyroidism itself.

In conclusion, herein we report the first case of painless thyroiditis with a significant and temporary increase of serum sIL-2R.

## Consent

Written informed consent was obtained from the patient for publication of this Case report and any accompanying images. A copy of the written consent is available for review by the Editor-in-Chief of this journal.

## Competing interest

The authors declare that they have no competing interests.

## Authors’ contributions

KH wrote the first draft. MY and KY were in a position of leadership for the patients and collected information on the patients. MK, HG and HK did the literature searches. SY wrote the final manuscript and made appropriate revisions. MI supervised the writing of the manuscript. All authors read through and approved the final manuscript.

## References

[B1] Cantrell DA Smith KA Transient expression of IL-2receptors. Conseqences for T cell growth J Exp Med 1983 6 1895 10.1084/jem.158.6.1895 6606011 PMC2187178

[B2] Rubin LA Nelson DL The soluble interleukin-2 receptor: biology, function and clinical application Ann Intern Med 1990 6 619 10.7326/0003-4819-113-8-619 2205142

[B3] Weryha G Gobert B Leclère J Béné MC Faure G Hartemann P Dynamic changes in soluble interleukin-2 receptor levels during treatment of Graves’ disease Horm Res 1991 6 8 12 10.1159/000181868 1916654

[B4] Balazs C Farid NR Soluble interleukin-2 receptor in sera of patients with Graves’ disease J Autoimmun 1991 6 4 681 688 10.1016/0896-8411(91)90185-F 1777014

[B5] Mariotti S Caturegli P Barbesino G Marinò M Del Prete GF Chiovato L Tonacchera M De Carli M Pinchera A Thyroid function and thyroid autoimmunity independently modulate serum concentration of soluble interleukin 2 receptor in thyroid diseases Clin Endocrinol (Oxf) 1992 6 5 415 422 10.1111/j.1365-2265.1992.tb02352.x 1486691

[B6] Intenzo CM Capuzzi DM Jabbour S Kim SM dePapp AE Scintigraphic features of autoimmune thyroiditis Radiographics 2001 6 4 957 964 10.1148/radiographics.21.4.g01jl17957 11452070

[B7] Ueda M Inaba M Kumeda Y Nagasaki T Hiura Y Tahara H Onoda N Ishikawa T Nishizawa Y The significance of thyroid blood flow at the inferior thyroid artery as a predictor for early Graves’ disease relapse Clin Endocrinol (Oxf) 2005 6 6 657 662 10.1111/j.1365-2265.2005.02397.x 16343100

[B8] Smallridge RC Tsokos GC Burman KD Porter L Cranston T Sfikakis PP Solomon BL Soluble interleukin-2 receptor is a thyroid hormone-dependent early-response marker in the treatment of thyrotoxicosis Clin Diagn Lab Immunol 1997 6 583 586 9302209 10.1128/cdli.4.5.583-586.1997 PMC170601

[B9] Koukkou E Panayiotidis P Thalassinos N Serum soluble interleukin-2 receptors as an index of the biological activity of thyroid hormones in hyperthyroidism J Endocrinol Invest 1995 6 4 253 257 7560805 10.1007/BF03347809

[B10] Ohtsuka R Abe Y Shiratsuchi M Suehiro Y Karube K Muta K Nishimura J Takayanagi R Graves’ disease with splenomegaly and pancytopenia, mimicking B-cell lymphoproliferative disease Rinsho Ketsueki 2008 6 2 104 108 18341041

[B11] Sellin JH Vassilopoulou-Sellin R Braverman LE, Utiger RD The gastrointestinal tract and liver in thyrotoxicosis Werner&Ingbar’s The Thyroid 2000 8 Philadelphia: Lippincott Williams & Wilkins 623 624

[B12] Nobili V Liaskos C **Autoimmune thyroiditis associated with autoimmune hepatitis** Thyroid 2005 6 10 1193 1195 10.1089/thy.2005.15.1193 16279855

[B13] Doniach D Roitt IM Walker JG Sherlock S Tissue antibodies in primary biliary cirrhosis, active chronic (lupoid) hepatitis, cryptogenic cirrhosis and other liver disease and their clinical implications Clin Exp Immunol 1966 6 3 237 262 5330183 PMC1579190

